# The effect of postoperative intravenous iron in anaemic, older cardiac surgery patients on disability-free survival (AGE ANEMIA study): study protocol for a multi-centre, double-blind, randomized, placebo-controlled trial

**DOI:** 10.1186/s13063-023-07725-y

**Published:** 2023-10-26

**Authors:** R. M. Smoor, T. C. D. Rettig, L. M. Vernooij, E. M. Groenewegen, H. P. A. van Dongen, P. G. Noordzij

**Affiliations:** 1 Department of Anaesthesiology, Intensive Care and Pain Medicine, St. Antonius Hospital, Nieuwegein, The Netherlands; 2 Department of Anaesthesiology, Intensive Care and Pain Medicine, Amphia Hospital, Breda, The Netherlands; 3 Department of Intensive Care Medicine, University Medical Centre Utrecht, Utrecht, The Netherlands

**Keywords:** Randomized controlled trial, Iron deficiency anaemia, Cardiac surgery, Elderly patients, Intravenous iron, Disability

## Abstract

**Background:**

Postoperative anaemia is common in older cardiac surgery patients and often caused by iron deficiency. Anaemia may negatively affect recovery after cardiac surgery. This study aims to determine the efficacy of treatment of postoperative iron deficiency anaemia (IDA) with intravenous iron (IVI) on disability 90 days after cardiac surgery in older patients.

**Methods:**

This is a randomized placebo-controlled double-blind multi-centre trial. In total, 310 patients aged ≥ 70 years with moderate IDA on postoperative day 1 (haemoglobin 85–110 g/L and ferritin concentration < 100 μg/L or iron saturation < 20%) after uncomplicated elective cardiac surgery (aortic valve repair or coronary artery bypass graft surgery) will be included. Patients will be randomly allocated to receive either IVI (ferric derisomaltose) or placebo (sodium chloride 0.9%) on postoperative day 1 in a 1:1 ratio, stratified by centre and type of cardiac surgery. The primary outcome is disability measured by the 12-item World Health Organization Disability Assessment score 2.0 after 90 days. Secondary outcome measures are the number of postoperative red blood cell (RBC) transfusions, change in reticulocyte haemoglobin content (pg) from randomization to hospital discharge, Hb levels at discharge, hospital complications, dyspnoea (assessed with the Rose Dyspnoea Scale) and health-related quality of life (HRQL) (assessed with The Older Persons and Informal Caregivers-Short Form (TOPICS-SF) questionnaire) after 90 days and days alive and out of hospital after 90 days. Lastly, the functional outcomes (e.g. steep ramp or 6-min walk test) and Hb level after 90 days will be assessed as an exploratory endpoint.

**Discussion:**

The results of this study will demonstrate whether early treatment of postoperative IDA with IVI improves disability at 90 days in older cardiac surgery patients.

**Trial registration:**

ClinicalTrials.gov NCT04913649. Registered on June 4, 2021.

**Supplementary Information:**

The online version contains supplementary material available at 10.1186/s13063-023-07725-y.

## Administrative information

Note: The numbers in curly brackets in this protocol refer to the SPIRIT checklist item numbers. The order of the items has been modified to group similar items (see http://www.equator-network.org/reporting-guidelines/spirit-2013-statement-defining-standard-protocol-items-for-clinical-trials/).

**Table:** 

Title {1}	Intravenous iron to treat postoperative anaemia in older cardiac surgery patients (AGE ANAEMIA study): study protocol for a multi-centre, double-blind, randomized, placebo-controlled trial
Trial registration {2a and 2b}	Trial identifier: NCT04913649, registered at ClinicalTrials.gov. All items from the WHO trial registry dataset can be found in the protocol.
Protocol version {3}	March 22^nd^ 2022, Version 2.
Funding {4}	Financial funding by Pharmacosmos A/S and St. Antonius Onderzoeksfonds
Author details {5a}	^1^Department of Anaesthesiology, Intensive Care and Pain medicine, St. Antonius Hospital. ^2^Department of Anaesthesiology, Intensive Care and Pain Medicine, Amphia Hospital. ^3^Department of Intensive Care Medicine, University Medical Centre Utrecht.
Name and contact information for the trial sponsor {5b}	St. Antonius Hospital Koekoekslaan 1 3435 CM Nieuwegein, The Netherlands T: + 31 (0) 883203000
Role of sponsor {5c}	This is an investigator-initiated clinical trial. The funders played no part in the design of the study; collection, analysis, and interpretation of the data; or writing of the manuscript.

## Introduction

### Background and rationale {6a}

Postoperative anaemia occurs in 80–90% of cardiac surgery patients due to blood loss, haemodilution, inflammation-induced blunted erythropoiesis and pre-existing anaemia [1]. The default treatment of postoperative anaemia is allogenic blood transfusion [2]. However, because of the associated risks with adverse outcomes, transfusion thresholds have become more restrictive over the years. This results in patients being discharged after surgery with low haemoglobin (Hb) levels [3]. In older non-surgical patients, anaemia is associated with disability, diminished physical performance and lower muscle strength [4, 5]. Furthermore, anaemic patients frequently suffer from fatigue, lethargy and dyspnoea [6, 7]. The negative effects of anaemia may impair physical functioning after cardiac surgery, which is a prognostic marker for mortality and readmission [8]. Elderly patients tolerate anaemia poorly and are at increased risk for poor functional outcomes due to frailty and multimorbidity [3, 9–11]. Especially in these patients, postoperative anaemia could impair postsurgical recovery, daily functioning and health-related quality of life (HRQL) [3, 9–11].

Although the underlying causes of postoperative anaemia are multifactorial, (functional) iron deficiency is present in the majority of the anaemic patients [1, 3, 12]. Oral iron supplementation is a recommended approach to treat iron deficiency anaemia (IDA), but treatment can take as long as 6 months to fully normalize Hb levels. Also, in post-surgical patients, the residual iron supplement remains largely unabsorbed in the digestive tract, leading to gastrointestinal side effects. As an alternative, intravenous iron (IVI) has yielded promising results in a number of surgical settings, and the positive effect of IVI on Hb levels remained present up to 6 months after treatment, although the evidence remains weak for cardiac surgery [12–17]. This suggests that IVI treatment might especially be beneficial in the recovery period. Prior studies on postoperative anaemia and functional outcome are scarce. In anaemic, nonsurgical patients with heart failure, treatment with IVI was proven beneficial regarding hospitalizations, quality of life scores and exercise capacity. It is plausible that anaemia correction with IVI can improve functional outcomes following cardiac surgery, especially in elderly patients. However, the effect of treatment of IDA on functional status after cardiac surgery in older patients has not yet been evaluated. We hypothesize that postoperative treatment of IDA with IVI improves functional outcomes in older patients after cardiac surgery.

### Objectives {7}

The primary objective is to evaluate the effect of early treatment of postoperative IDA with intravenous iron (IVI) compared to placebo on disability after 90 days in older patients after elective cardiac surgery. The secondary objectives are to evaluate whether administration of postoperative IVI improves patient-reported outcome measures (PROMs) related to dyspnoea symptoms and health-related quality of life (HRQL) after 90 days. Additionally, we will investigate the effect of treatment with IVI on postoperative RBC transfusions, change in reticulocyte haemoglobin content (pg) during hospitalization, Hb levels at discharge, hospital complications and days alive and out of hospital after 90 days. Lastly, functional outcomes (e.g. steep ramp or 6-min walk test) and Hb levels after 90 days will be assessed as an exploratory endpoint.

### Trial design {8}

This is a randomized placebo-controlled double-blind multi-centre superiority trial with two parallel groups in which IVI is compared to a placebo. The patient allocation ratio is 1:1, stratified by centre and type of cardiac surgery.

## Methods: participants, interventions and outcomes

### Study setting {9}

This study is initiated in two large community hospitals in the Netherlands (St. Antonius Hospital, Nieuwegein, and Amphia Hospital, Breda). Patients are preoperatively recruited and eligible if they meet the criteria as defined below.

Primary inclusion criteria:

In order to be eligible to participate in this study, a subject must meet all of the following criteria:

- Age ≥ 70 years and mentally competent
- Elective AVR or CABG surgery (including AVR or CABG surgery combined with rhythm surgery, i.e. maze, pulmonary vein isolation and left atrial appendage closure)

Primary exclusion criteria:

A potential subject who meets any of the following criteria will be excluded from participation in this study:

- Medical history of iron overload/haemochromatosis
- Medical history of liver cirrhosis or ALT/AST serum concentrations > 3 times the reference value (female patients: ALT > 120, AST > 105 U/L, male patients: ALT > 150, AST > 135 U/L)
- Renal failure (eGFR < 15 mL/min/1.73 m^2^)
- Recent treatment with IVI (< 12 weeks prior to surgery date)
- Serious or severe allergic reaction to IVI in medical history
- Severe asthma or eczema in medical history (atopic constitution)

Definitive eligibility is assessed at postoperative day (POD) 1 based on the criteria below:

1. Moderate postoperative IDA, defined as:
  - Hb between 85 and 110 g/L
  - (TSAT) < 20%
2. Meeting the criteria for discharge from ICU to general ward (uncomplicated surgery), defined as:
  - No inotropic agents or ventilation at the time of final inclusion (POD 1)
  - Expected discharge to a general ward at POD 1

### Who will take informed consent? {26a}

Eligible patients will be informed about this study by means of a conversation with the anaesthesiologist and an information brochure. The aims of the study, the methods used and the potential risks and benefits for the patient are described in this brochure. Patients are given the opportunity to ask questions about the study during and after visiting the outpatient clinic. After a reasonable term, the patients will be contacted by telephone for informed consent by a member of the research team. At hospital admission, informed consent will be signed by the patient and study personnel.

### Additional consent provisions for collection and use of participant data and biological specimens {26b}

Not applicable. No biological specimens or additional data will be collected.

## Interventions

### Explanation for the choice of comparators {6b}

The control group will receive 250-mL sodium chloride (NaCl) 0.9% as an infusion drip (placebo). This will ensure an optimal comparison with the treatment group.

### Intervention description {11a}

The patient will undergo surgery according to standard practice. Postoperatively, patients will be admitted to the ICU (standard care). On the first postoperative morning, the iron status will be assessed from a routine blood sample in patients with uncomplicated surgery who meet the ICU discharge criteria. Patients with IDA will be randomly allocated to either the treatment group or the placebo group. Patients randomized to the treatment arm will be administered a single dose of ferric derisomaltose (Monofer®) 100 mg/mL solution for infusion in 60 min. The method of administration and dosage of the investigational medication are standard treatment. The ferric derisomaltose dose will be calculated for each patient depending on ideal body weight (20 mg/kg) and diluted in 250 mL NaCl 0.9%. The maximum ferric derisomaltose dose is 2000 mg. The placebo group will receive 250 mL NaCl 0.9% w/v in 60 min. The study medication will be administered before ICU discharge by a trained nurse. Vital signs of the patient will be monitored during administration of the study medication and for 30 min afterwards. The study medication will be administered through a central venous catheter which is placed after induction of anaesthesia before the start of surgery (standard care). No additional venous access is required to administer the study medication. Both the infusion bag and the infusion line are light-protected (Codan®). Participation in the trial will not delay ICU discharge.

### Criteria for discontinuing or modifying allocated interventions {11b}

Patients can leave the study at any time for any reason without any consequences. The patient data that have been collected up to that moment will be included in the analysis. Acute severe hypersensitivity reactions may occur with parenteral iron preparations. They usually occur within the first few minutes of administration and are generally characterized by the sudden onset of respiratory difficulty and/or cardiovascular collapse. In the case of signs/symptoms of acute hypersensitivity reactions or anaphylactic shock (frequency is rare: incidence is less than 0.1%), the intervention will be discontinued and the patient will be excluded from the study. An excluded patient will be replaced by a new patient. Hypersensitivity reactions and anaphylactic shock will be treated according to standard protocol. In addition, a Fishbane reaction may occur in patients treated with IVI. A Fishbane reaction is characterized by flushing of the face, acute chest and/or back pain and tightness in the chest, which is sometimes accompanied by dyspnoea. This may mimic the early symptoms of an anaphylactic reaction. However, they disappear shortly after administration is discontinued. Approximately 15 min after the symptoms have disappeared, the infusion will be restarted with a 50% lower infusion rate. A Fishbane reaction typically does not reoccur if IVI administration is restarted at a lower infusion rate and is therefore not necessarily a reason to exclude the patient from the study.

In accordance to Sect. 10, subsection 4, of the Dutch Medical Research with Human Subjects Law (WMO (Wet medisch-wetenschappelijk onderzoek met mensen)), the sponsor (St. Antonius Hospital) should suspend the study if there are sufficient grounds that continuation of the study will jeopardize subject health or safety. However, ferric derisomaltose is an IV therapy approved by the EMA. The EMA concluded that the benefit-risk balance of intravenous iron is favourable in the treatment of iron deficiency when used under their current indications. In this study, ferric derisomaltose will be used within its approved indication, and subsequently, the risks associated with the treatment are considered low.

### Strategies to improve adherence to interventions {11c}

Apart from the intervention at POD 1, patients will receive standard care during hospital admission. Prior to the start of the trial, nurses and physicians at the ICU have received information about the study protocol and were trained in the administration of IVI infusion and handling of infusion reactions, which will improve protocol adherence.

### Relevant concomitant care permitted or prohibited during the trial {11d}

Not applicable. No relevant concomitant care is prohibited during the trial.

### Provisions for post-trial care {30}

The sponsor has a trial subject insurance for patients participating in the study. The insurance provides coverage for study-related damage, which becomes apparent during or within 4 years after the end of the study. Participants receive information on the trial subject insurance in the study information brochure.

### Outcomes {12}

The primary endpoint is the difference in median disability scores as measured by the 12-item World Health Organization Disability Assessment score 2.0 (12-item WHODAS 2.0) 90 days after elective cardiac surgery, between the two treatment arms. The 12-item WHODAS 2.0 is a validated questionnaire for the assessment of postsurgical disability in the following domains: communication, mobility, self-care, interacting with other people, taking care of housework and participating in the community. A disability score is calculated ranging from 0% (no disability) to 100% (fully disabled, including death). The difference in the proportion of patients with new disability between the two treatment arms is a secondary endpoint, defined as an increase in the 12-item WHODAS 2.0 score ≥ 5% at day 90 compared to the preoperative assessment (baseline) [18]. Other secondary endpoints are the difference in the median number of red blood cell (RBC) transfusion administered during hospital stay, the median change in reticulocyte haemoglobin content from POD 1 to hospital discharge, median Hb levels at discharge, proportion of in-hospital complications, median days alive and out of the hospital after 90 days, median score regarding dyspnoea symptoms after 90 days (assessed with the Rose Dyspnoea Scale (RDS)) and median HRQL scores (assessed with The Older Persons and Informal Caregivers-Short Form (TOPICS-SF) questionnaire) after 90 days. Lastly, we will consider functional outcomes (e.g. steep ramp or 6-min walk test) and median Hb levels after 90 days, as exploratory endpoints.

### Participant timeline {13}

**Table:** 

**Participant timeline**	
**Outpatient clinic**	
Patient is provided with study information	
**Pre-surgery hospital admission**	
Written informed consent	
Baseline blood values are collected (from a routine preoperative blood sample)	
Baseline PROMs (WHODAS-12, TOPICS-SF, RDS) are assessed	
**Surgery**	
Patient is routinely admitted to the ICU after surgery	
**Postoperative day 1**	
Blood sample (iron status, ret Hb count)	
Definite inclusion in case of IDA	
Randomization	
Intervention:	
Group A: IVI	Group B: placebo
**Hospital discharge (± 5 days postoperative)**	
Blood sample (ret Hb count, Hb level)	
**90 days after surgery**	**90 days exploratory endpoints**
Follow-up PROMs (WHODAS-12, TOPICS-SF, RDS)	Results from the functional tests (steep-ramp/6-min walk test) Hb level

### Sample size {14}

In the previously conducted AGE cohort (ClinicalTrials.gov Identifier NCT02535728) studying a similar patient population, the mean WHODAS 2.0 score for CABG and AVR patients after 3 months was 12% points with a standard deviation (SD) of 15% points. For an expected effect size, a mean difference in WHODAS 2.0 score of 5% points or more between the treatment and placebo groups was chosen. This difference in WHODAS 2.0 score is consistent with a clinically relevant change in disability [18]. We based our sample size estimate on the most conservative (largest) standard deviation, which was 15% points. To detect a mean difference in WHODAS 2.0 score of 5% points at 90 days using a two-tailed unpaired *T*-test with a two-sided significance level of 5% and a power of 80% with equal allocation to two arms would require 142 patients per treatment group. Taking into account a loss to follow-up margin of 10%, we aim to include a total cohort of 310 patients. Calculations were performed using R version 3.6.1—© 2019–07-05, R, Inc., for Windows.

### Recruitment {15}

Patients will be recruited at the preoperative assessment outpatient clinic of the participating hospitals by providing them with oral information and folders with study information.

## Assignment of interventions: allocation

### Sequence generation {16a}

Via a computer-generated randomization (randomization list will be generated using R version 3.6.1—© 2019–07-05, R, Inc., for Windows), patients will be randomized, stratified by hospital site and surgical intervention (AVR or CABG).

### Concealment mechanism {16b}

After the inclusion of the patient, the randomization and preparation of the trial medication will be overseen by a clinical pharmacist. Both participant and researcher will be blinded for the trial medication. As the ferric derisomaltose solution is brown and the placebo solution is colourless, the infusion bags and lines will be light-protected in order to prevent identification of the investigational product. The study medication will be administered through the central venous line, which is placed in the jugular vein in the neck and therefore out of sight for the patient. The patient’s general practitioner will be informed with regard to the study participation of the patient.

### Implementation {16c}

After signing the informed consent, research personnel that are not blinded for the allocation treatment will enrol the patients and use REDCap (VanderBilt University) to allocate the patient to one of the study arms. The clinical pharmacist will receive an allocation list that is automatically generated by REDCap (using an allocation sequence that was generated prior the trial by a member of the research team (RS)) with patients’ study IDs and corresponding treatment allocation and will prepare the appropriate trial medication.

## Assignment of interventions: blinding

### Who will be blinded {17a}

The members of the research team that will assess the outcome and analyse the data, as well as the patients will be blinded for the allocated intervention.

### Procedure for unblinding if needed {17b}

In the case of a suspected unexpected serious adverse reaction (SUSAR), the principle investigator is permitted to request a code break for the unblinding of the intervention allocation. The clinical pharmacist will perform the code break.

## Data collection and management

### Plans for assessment and collection of outcomes {18a}

Data will be derived from electronic patient records and collected with an electronic case report form (eCRF) using REDCap. Laboratory tests are performed by the clinical laboratory. Patients can fill out the baseline and follow-up questionnaires either digitally (the answers will be directly saved in REDCap) or on paper. If patients fill out the questionnaires on paper (Supplementary file 1), a member of the research team will enter the information manually into REDCap. For the exploratory endpoints, the results from functional tests (steep ramp or 6-min walk test) measured by the physical therapist as part of routine postoperative care are requested. In addition, laboratory results (i.e. Hb level if available) from outpatient visits for postoperative follow-up with the treating cardiologist are requested as an exploratory endpoint. All research personnel will be trained in the study requirements. All data outcome definitions are reported in a data management manual and a codebook, ensuring that the collected data is standardized.

### Plans to promote participant retention and complete follow-up {18b}

The patients will receive extensive information about the study set-up and requirements during the recruitment. The importance of completion of follow-up will be stressed. After 90 days, the patients are asked to fill out an electronic questionnaire (if possible) in REDCap. REDCap automatically generates reminders if patients do not respond to the request to fill out the questionnaire. If patients are not able to fill out the questionnaire electronically, the questionnaires will be sent by mail with a prepaid return envelope. Throughout the follow-up period, the researchers will check responses and if necessary contact patients for completion of their follow-up.

### Data management {19}

All data will be collected by the investigators and handled in a study database (REDCap) that is protected by two-factor authentication and only accessible to investigators of the study. REDCap is a secure web application for building and managing databases. To avoid erroneous values, a data range will be provided for all continuous data values. Questionnaires will be either filled out electronically by the patient in REDCap or entered manually by the investigators. Informed consent and end-of-trial dates will be recorded in the electronic patient dossier and signed paper forms will be stored within the hospitals in a locked cabinet. All changes to the raw data in REDCap are automatically recorded. All steps taken in the analysis will be documented in R (version 3.5.1—© 2018–07-02, R, Inc., for Windows). The dataset will be kept for 15 years.

### Confidentiality {27}

Patients will be assigned a unique study ID that is comprised of 2 letters (AN) and 3 digits (AN001, AN002, etc.). Personal information from study subjects will be untraceable from this study ID. A file containing the surname, date of birth and the corresponding study ID of all study patients will be safeguarded on a secure, password-protected hard drive on a hospital computer by the principal investigator of each participating hospital. All patient information will be processed according to the EU General Data Protection Regulation and the Dutch Act on Implementation of the General Data Protection Regulation (AVG).

### Plans for collection, laboratory evaluation and storage of biological specimens for genetic or molecular analysis in this trial/future use {33}

Not applicable. No biological specimens are collected.

## Statistical methods

### Statistical methods for primary and secondary outcomes {20a}

Data will be analysed using R statistics (version 3.5.1—© 2018–07-02, R, Inc., for Windows). Descriptive statistics will be calculated for all study parameters. Continuous data will be described as mean (+ standard deviation) and median (+ interquartile range) for normally and non-normally distributed data, respectively. Categorical variables will be described as numbers and percentages. The effect of IVI therapy on the primary and secondary endpoints will be analysed with ANCOVA and logistic regression analysis. All analyses are performed according to the intention-to-treat principle. Two-sided *P* values of 0.05 or less will be considered statistically significant.

Baseline characteristics will be described per treatment arm as percentages, means (± SDs) or medians (IQRs) as appropriate. The primary outcome, disability as measured by WHODAS-12 scores after 90 days will be compared between the two groups with the independent Student’s *t* test or Wilcoxon rank sum test, depending on the distribution of the data. Thereafter, to evaluate the effect of IVI on the primary outcome, an ANCOVA analysis will be performed with an adjustment for baseline Hb levels and WHODAS-12 scores. Effect estimates are presented as *β* with accompanying 95% confidence intervals (CI). New disability, defined as an increase in the WHODAS score ≥ 5% from baseline, will be compared between the two treatment groups by logistic or Poisson regression analysis and expressed with odds ratio (OR) or relative risks (RR), if appropriate. For the secondary outcomes, the difference in dyspnoea from baseline, assessed with the RDS, difference in Hb from baseline, difference in HRQL from baseline and number of red blood cell transfusions will be summarized descriptively and compared between the treatment groups with the independent Student’s *t* test or Wilcoxon rank sum test, depending on the distribution of the data. The difference in median days alive and out of the hospital at 90 days between the treatment groups will be analysed with the Wilcoxon rank sum test. The effect of IVI on secondary outcomes will be evaluated with ANCOVA analysis. Postoperative complications will be summarized by frequency and percentage and compared in the treatment groups with logistic regression analysis or Poisson regression analysis. As an exploratory endpoint, the results from functional capability tests (steep-ramp or 6-min walk test) after 90 days will be evaluated if available. The effect of postoperative IVI on functional capability tests will be evaluated with logistic or linear regression analysis, where appropriate. Another exploratory endpoint is a difference in Hb level after 90 days. The independent Student’s *t* test or the Wilcoxon rank sum will be used to compare Hb levels between the two treatment arms and linear regression analysis will be performed to evaluate the effect between the groups.

### Interim analyses {21b}

Not applicable. No interim analysis will be performed.

### Methods for additional analyses (e.g. subgroup analyses) {20b}

For sensitivity analysis, baseline differences between treatment arms are assessed with the independent Student’s *t* test or Wilcoxon rank sum test and if needed, multivariable analyses will be used to adjust for possible imbalances. Subgroup analysis will be performed for CABG and AVR surgery patients separately.

### Methods in analysis to handle protocol non-adherence and any statistical methods to handle missing data {20c}

We intend to test superiority using an intention-to-treat analysis. Thus, all randomized participants, regardless of protocol adherence, as randomized to treatment allocation will be included in the analysis. Missing data will be reduced to a minimum, as described above. However, in case of missing data, these will be compared between the two groups, and imputed using multiple imputations.

### Plans to give access to the full protocol, participant-level data and statistical code {31c}

The datasets used and/or analysed during the current study can be made available by the corresponding author upon reasonable request and in agreement with the research collaboration and data transfer guidelines of the participating hospitals.

## Oversight and monitoring

### Composition of the coordinating centre and trial steering committee {5d}

This is a multicentre study, initiated in two hospitals.

For day-to-day support, the following roles are assigned:

- The coordinating investigator is responsible for trial registration, the coordination of study visits and the communication between participating hospitals and annual safety reports.
- The principal investigators in the participating hospitals supervise the trial and the medical responsibility of the patients. They are responsible for data management (organizing data capture, safeguarding quality and data). Furthermore, the principal investigators are responsible for patients’ recruitment, the attainment of informed consent and follow-up.

The study team meets twice a month. There is no Trial Steering Committee or stakeholder and public involvement group.

### Composition of the data monitoring committee, its role and reporting structure {21a}

A data safety monitoring board (DSMB) has not been appointed for this study since the risk classification for this study is low. The study population (elderly cardiac surgery patients) is considered a high-risk population. The intervention, however, is considered a low-risk intervention because of the following reasons:

- First, the study drug is approved by the EMA in the EU in 2009 and is already a common treatment for IDA.
- Second, the study drug will be used within its approved indication in the study, and subsequently, the risks associated with the treatment are considered low. Third, the patient’s vital signs are continuously monitored during and after treatment (at the ICU). Lastly, even though there is a small risk for acute severe hypersensitivity reactions (an anaphylactic reaction is rare (incidence is < 0.1%)), the EMA concluded that the benefit-risk balance of IVI treatment is favourable as the benefits outweigh the risks in the treatment of iron deficiency when used under their current indications.

### Adverse event reporting and harms {22}

Adverse events (AEs) are defined as any undesirable experience occurring to a subject during the study, whether or not considered related to the investigational product. All AEs reported spontaneously by the subject or observed by the investigator or staff will be recorded. All AEs will be evaluated for severity, seriousness and relatedness to the investigational product. A serious adverse event (SAE) is any untoward medical occurrence or effect that:

- Results in death
- Is life-threatening (at the time of the event)
- Requires hospitalization or prolongation of existing inpatient’s hospitalization
- Results in persistent or significant disability or incapacity
- Is a congenital anomaly or birth defect
- Any other important medical event that did not result in any of the outcomes listed above due to medical or surgical intervention but could have been based upon appropriate judgement by the investigator

The investigator will report all SAEs to the sponsor without undue delay after obtaining knowledge of the events, except for the SAEs which are common after cardiac surgery and do not require immediate reporting, such as cardiogenic shock (> 1 h after infusion), (supra)ventricular arrhythmia, atrioventricular block, congestive heart failure, acute kidney injury, pneumonia, pulmonary embolus, stroke, sepsis, wound infection, urinary tract infection, delirium, reoperation and ICU readmission (from general ward). The SAEs described above will be recorded in an overview list (line-listing) that will be submitted once every half year to the Medical Ethical Committee. This line-listing provides an overview of all SAEs, accompanied by a brief report highlighting the main points of concern. All other SAEs will be reported to the CCMO following the CCMO guidelines.

### Frequency and plans for auditing trial conduct {23}

As this is an investigator-initiated study, an independent study monitor will be appointed by the research and development (R&D) department from the St. Antonius Hospital for study-specific auditing. The independent monitor checks the presence and completeness of the investigation file. Moreover, the monitor checks the informed consents, inclusion and exclusion criteria, source data, and missing and reporting for (S)AEs/SUSARs.

### Plans for communicating important protocol amendments to relevant parties (e.g. trial participants, ethical committees) {25}

Amendments are changes made to the research after a favourable opinion by the accredited medical ethical committee (METC) has been given. All amendments will be notified to the METC that gave a favourable opinion. A ‘substantial amendment’ is defined as an amendment to the terms of the METC application, or to the protocol or any other supporting documentation, that is likely to affect to a significant degree:

- The safety or physical or mental integrity of the subjects of the trial
- The scientific value of the trial
- The conduct or management of the trial
- The quality or safety of any intervention used in the trial

All substantial amendments will be notified to the METC and to the competent authority. Non-substantial amendments will not be notified to the accredited METC and the competent authority but will be recorded and filed by the sponsor.

### Dissemination plans {31a}

Subjects are entitled to public disclosure of the results of the trial based on their participation. The results of this research will be disclosed completely in international peer-reviewed journals. Both positive and negative results will be reported.

## Discussion

The use of IVI as perioperative therapy has been studied in other surgical subspecialties with promising results; A recent clinical trial in abdominal surgery reported that IVI given preoperatively may reduce readmissions for complications in the postoperative period. A systematic review in 2019 showed that the current evidence for IVI preoperatively in orthopaedic surgery patients supports its use to decrease the number of transfusions, length of stay, and infection. Postoperative IVI treatment also resulted in higher Hb levels after 4 weeks as well as lower transfusion rates in elective non-cardiac surgery patients, compared to a placebo [12, 13, 15, 16]. However, the data on the use of IVI for cardiac surgery patients are less robust due to the limited number of RCTs and a lack of adequately powered studies [17, 19]. In cardiac surgery patients are at an increased risk for anaemia. The negative effects of anaemia may impair physical functioning and recovery. However, whether postoperative IVI treatment can improve recovery and disability-free survival is yet to be determined. This randomized controlled trial aims to evaluate the effect of postoperative IVI treatment on disability in elderly cardiac surgery patients with moderate IDA. Potentially, this could lead to the implementation of routine IVI treatment in anaemic patients after cardiac surgery. However, there are some limitations to consider. First of all, the primary outcome of this trial is self-assessed disability after 90 days. For this reason, it is imperative that the participating subjects are completely blinded for the study medication. Ferric derisomaltose is brown and the placebo colourless. Even though the infusion bags and lines will be light-protected, incomplete concealment can occur which could potentially influence the participant. Although rare, an infusion reaction to IVI treatment may also break concealment. That being said, all measures are taken to ensure the complete blinding of all participants. Therefore, we feel that the level of evidence of this trial will be higher than an open-label trial. Second, to provide exploratory data on the effect of postoperative IVI treatment on erythropoiesis, Hb levels will be requested from the treating cardiologist after 90 days as an exploratory endpoint. As this information will not be available for all participants, data will be incomplete. Also, Hb levels are likely tested in patients with a clinical indication, leading to bias. However, we feel that an additional blood draw after 90 days adds complexity to our pragmatic trial design and will increase loss of follow-up and lower of participants.

Major strengths of this trial are its pragmatic nature, multi-centre, double-blinded, placebo-controlled design and extensive outcome parameters. We expect to provide valuable and definite answers on the effect of IVI on postoperative disability, quality of life and recovery in the elderly cardiac surgery patient.

## Trial status

Recruitment started in November 2021. The current protocol is version two of 22–03-2022. Currently, we included thirty patients. Patient recruitment is estimated to be completed around December 2023.

### Supplementary Information

**Additional file 1.**

## Data Availability

The datasets used during the current study will be made available from the corresponding author (PN) upon reasonable request, who will have access to the final trial dataset.

## Abbreviations

AVR: Aortic valve replacement
CABG: Coronary artery bypass graft
CCMO: Central Committee on Research Involving Human Subjects
DSMB: Data Safety Monitoring Board
eCRF: Electronic case report form
HRQL: Health-related quality of life
IDA: Iron deficiency anaemia
IVI: Intravenous iron
METC: Medical Ethical Committee
POD: Postoperative day
RBC: Red blood cell
RDS: Rose Dyspnoea Scale
SAE: Serious adverse event
SUSAR: Suspected unexpected serious adverse reaction
TOPICS-SF: The Older Persons and Informal Caregivers-Short Form
WHODAS-12: 12-Item World Health Organization Disability Assessment score 2.0

## References

[1] Gómez-Ramirez S, Jericó C, Muñoz M (2019). Perioperative anemia: prevalence, consequences and pathophysiology. Transfus Apher Sci Off J World Apher Assoc Off J Eur Soc Haemapheresis.

[2] Dhir A, Tempe DK (2018). Anemia and patient blood management in cardiac surgery-literature review and current evidence. J Cardiothorac Vasc Anesth.

[3] Muñoz M, Acheson AG, Bisbe E (2018). An international consensus statement on the management of postoperative anaemia after major surgical procedures. Anaesthesia.

[4] Penninx BWJH, Pahor M, Cesari M (2004). Anemia is associated with disability and decreased physical performance and muscle strength in the elderly. J Am Geriatr Soc.

[5] Han C, Rahman MU, Doyle MK (2007). Association of anemia and physical disability among patients with rheumatoid arthritis. J Rheumatol.

[6] Lind M, Vernon C, Cruickshank D (2002). The level of haemoglobin in anaemic cancer patients correlates positively with quality of life. Br J Cancer.

[7] Wouters HJCM, van der Klauw MM, de Witte T (2019). Association of anemia with health-related quality of life and survival: a large population-based cohort study. Haematologica.

[8] Cacciatore F, Abete P, Mazzella F (2012). Six-minute walking test but not ejection fraction predicts mortality in elderly patients undergoing cardiac rehabilitation following coronary artery bypass grafting. Eur J Prev Cardiol.

[9] Chaves PHM, Semba RD, Leng SX (2005). Impact of anemia and cardiovascular disease on frailty status of community-dwelling older women: the women’s health and aging studies I and II. J Gerontol A Biol Sci Med Sci.

[10] Gill TM, Allore HG, Holford TR (2004). Hospitalization, restricted activity, and the development of disability among older persons. JAMA.

[11] Covinsky KE, Palmer RM, Fortinsky RH (2003). Loss of independence in activities of daily living in older adults hospitalized with medical illnesses: increased vulnerability with age. J Am Geriatr Soc.

[12] Xu H, Duan Y, Yuan X (2019). Intravenous iron versus placebo in the management of postoperative functional iron deficiency anemia in patients undergoing cardiac valvular surgery: a prospective, single-blinded, randomized controlled trial. J Cardiothorac Vasc Anesth.

[13] Richards T, Baikady RR, Clevenger B (2020). Preoperative intravenous iron to treat anaemia before major abdominal surgery (PREVENTT): a randomised, double-blind, controlled trial. Lancet.

[14] Johansson PI, Rasmussen AS, Thomsen LL (2015). Intravenous iron isomaltoside 1000 (Monofer®) reduces postoperative anaemia in preoperatively non-anaemic patients undergoing elective or subacute coronary artery bypass graft, valve replacement or a combination thereof: a randomized double-blind placebo-. Vox Sang.

[15] Khalafallah AA, Yan C, Al-Badri R (2016). Intravenous ferric carboxymaltose versus standard care in the management of postoperative anaemia: a prospective, open-label, randomised controlled trial. Lancet Haematol.

[16] van Veldhuisen DJ, Ponikowski P, van der Meer P (2017). Effect of ferric carboxymaltose on exercise capacity in patients with chronic heart failure and iron deficiency. Circulation.

[17] Tankard KA, Park B, Brovman EY (2020). The impact of preoperative intravenous iron therapy on perioperative outcomes in cardiac surgery: a systematic review. J Hematol.

[18] Shulman MA, Kasza J, Myles PS (2020). Defining the minimal clinically important difference and patient-acceptable symptom state score for disability assessment in surgical patients. Anesthesiology.

[19] Kong R, Hutchinson N, Hill A (2022). Randomised open-label trial comparing intravenous iron and an erythropoiesis-stimulating agent versus oral iron to treat preoperative anaemia in cardiac surgery (INITIATE trial). Br J Anaesth.

