# Supplementary material for: The effect of postoperative intravenous iron in anaemic, older cardiac surgery patients on disability-free survival (AGE ANEMIA study): study protocol for a multi-centre, double-blind, randomized, placebo-controlled trial
Source: Trials. 2023 Oct 26;24:693. doi: 10.1186/s13063-023-07725-y (PMC10601172; doi:10.1186/s13063-023-07725-y)
Supplement: Supplementary file 1 — Additional file 1. [file 13063_2023_7725_MOESM1_ESM.docx]

**Baseline.**

**Studienummer:**

**Datum:**

U ontvangt deze vragenlijst omdat u meedoet aan de AGE ANEMIA trial. Het is van belang dat u de vragenlijsten zo compleet mogelijk invult.

Alvast hartelijk bedankt voor uw medewerking.


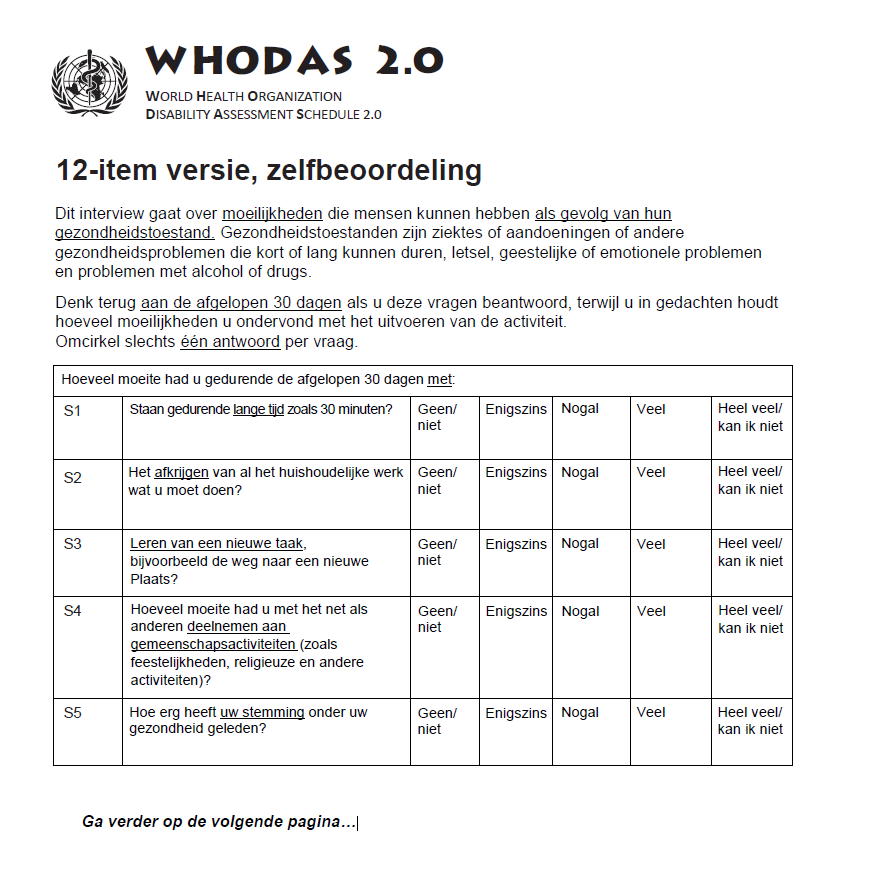


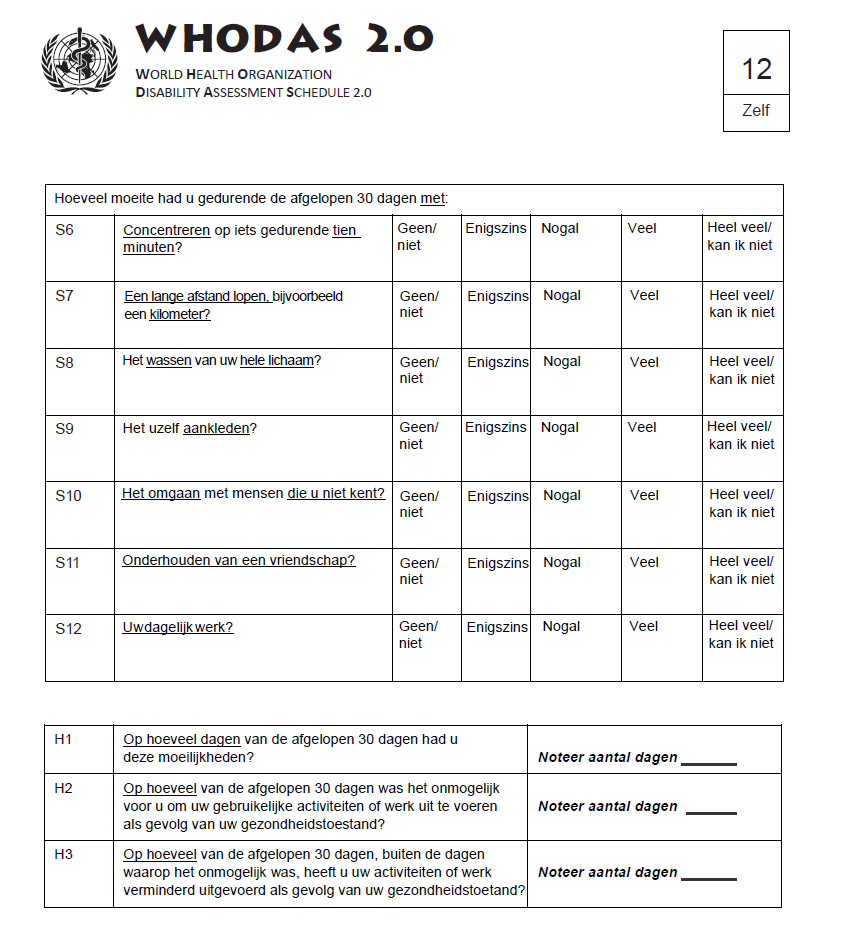


**Rose dyspneu schaal**

Omcirkel het antwoord welke het beste bij uw situatie past.

Bij het beantwoorden van de onderstaande vragen, denkt u terug aan hoe u zich **de afgelopen maand** heeft gevoeld:

| 1. Raakt u buiten adem als u zich erg moet haasten of als een lichte heuvel op loopt? | **JA**  **NEE** |
| --- | --- |
| 1. Raakt u buiten adem als u **samen met uw leeftijdsgenoten** loopt zonder daarbij trappen of heuvels te beklimmen? | **JA**  **NEE** |
| 1. Raakt u buiten adem als u **op uw eigen tempo** loopt zonder daarbij trappen of heuvels te beklimmen? | **JA**  **NEE** |
| 1. Raakt u buiten adem als u zichzelf wast of aankleedt? | **JA**  **NEE** |

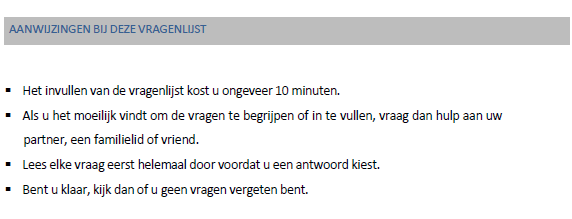


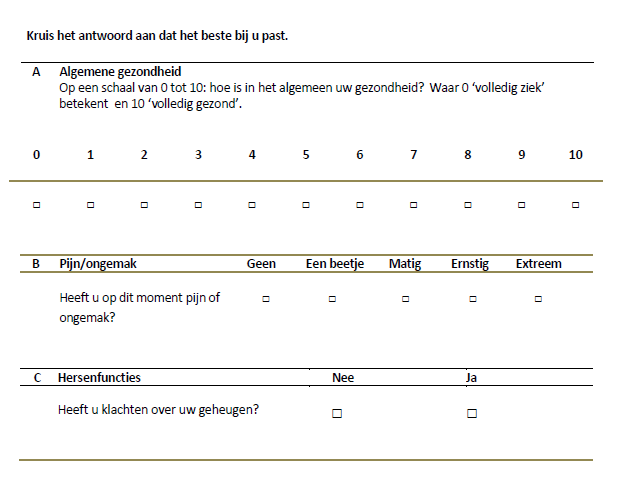


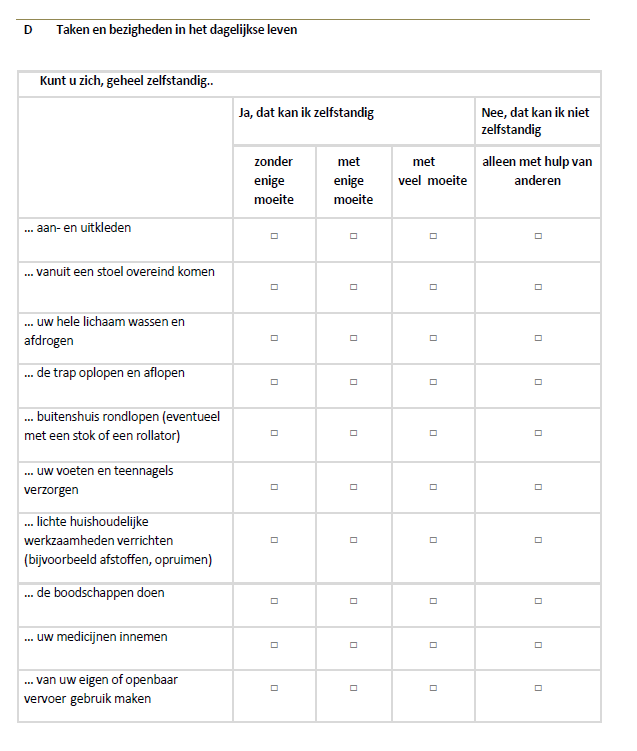


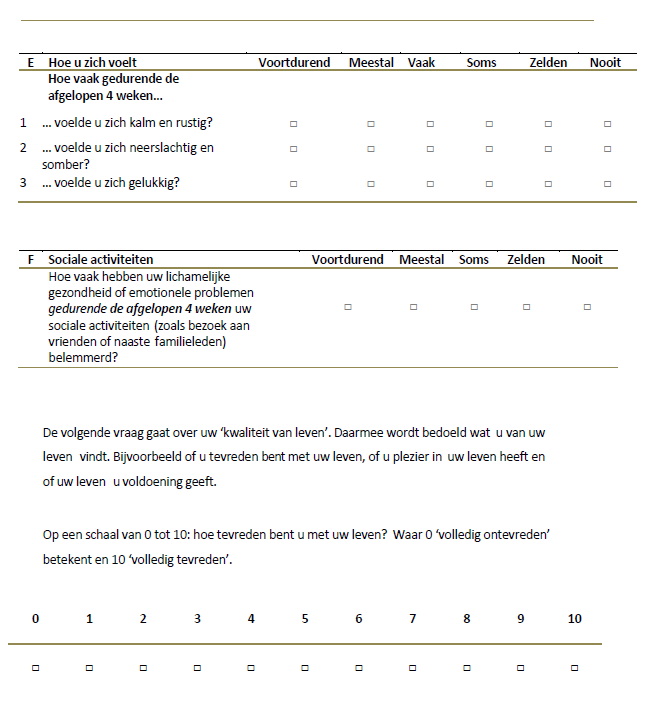


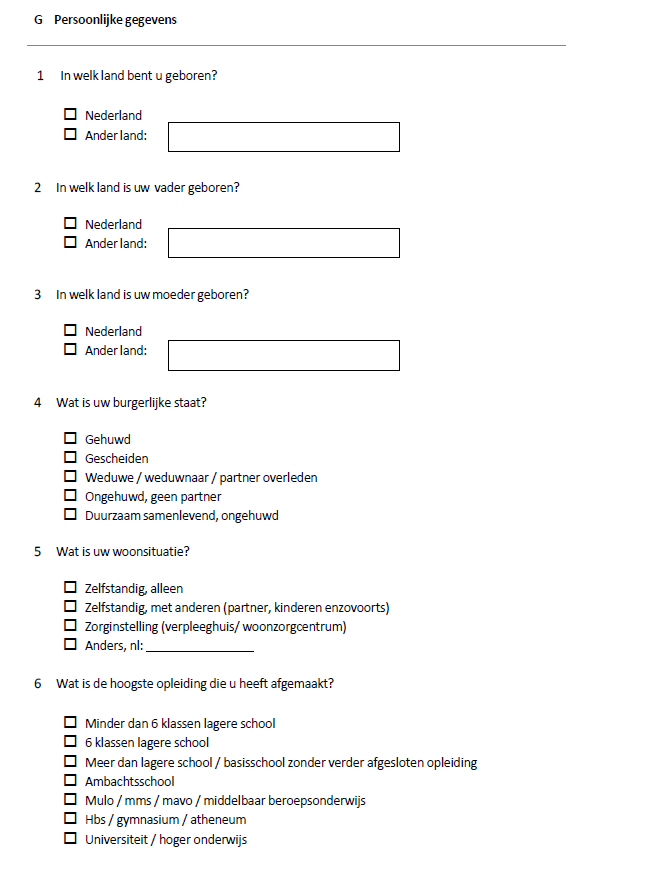


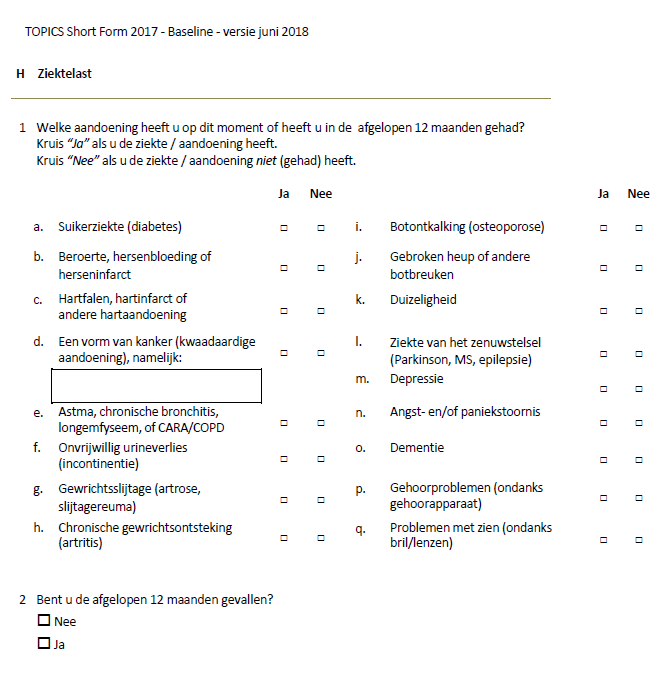


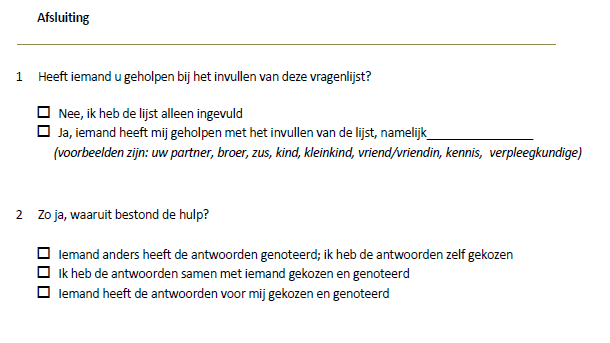


**Dit is het einde van de vragenlijst. Wij danken u hartelijk voor het invullen.**
